# Transcervical Ultrasonography Is Feasible to Visualize and Evaluate Base of Tongue Cancers

**DOI:** 10.1371/journal.pone.0087565

**Published:** 2014-01-30

**Authors:** Ray Gervacio F. Blanco, Joseph Califano, Barbara Messing, Jeremy Richmon, Jia Liu, Harry Quon, Geoffrey Neuner, John Saunders, Patrick K. Ha, Sheila Sheth, Maura Gillison, Carole Fakhry

**Affiliations:** 1 Department of Otolaryngology - Head and Neck Surgery, Division of Head and Neck Surgery, Johns Hopkins Medicine, Baltimore, Maryland, United States of America; 2 Milton J. Dance, Jr. Head and Neck Center, Greater Baltimore Medical Center, Baltimore, Maryland, United States of America; 3 Department of Radiation Oncology and Molecular Radiation Sciences, Johns Hopkins Medicine, Baltimore, Maryland, United States of America; 4 Department of Radiology, Greater Baltimore Medical Center, Baltimore, Maryland, United States of America; 5 Department of Radiology, Johns Hopkins Medicine, Baltimore, Maryland, United States of America; 6 Department of Internal Medicine, Ohio State University Comprehensive Cancer Center, Columbus, Ohio, United States of America; 7 Department of Epidemiology, Johns Hopkins Bloomberg School of Public Health, Baltimore, Maryland, United States of America; University of Pécs Medical School, Hungary

## Abstract

**Background:**

Base of tongue (BOT) is a difficult subsite to examine clinically and radiographically. Yet, anatomic delineation of the primary tumor site, its extension to adjacent sites or across midline, and endophytic vs. exophytic extent are important characteristics for staging and treatment planning. We hypothesized that ultrasound could be used to visualize and describe BOT tumors.

**Methods:**

Transcervical ultrasound was performed using a standardized protocol in cases and controls. Cases had suspected or confirmed BOT malignancy. Controls were healthy individuals without known malignancy.

**Results:**

100% of BOT tumors were visualized. On ultrasound BOT tumors were hypoechoic (90.9%) with irregular margins (95.5%). Ultrasound could be used to characterize adjacent site involvement, midline extent, and endophytic extent, and visualize the lingual artery. No tumors were suspected for controls.

**Conclusions:**

Ultrasonography can be used to transcervically visualize BOT tumors and provides clinically relevant characteristics that may not otherwise be appreciable.

## Introduction

The incidence of oropharyngeal squamous cell cancer (OPSCC) is rapidly rising in the United States and is expected to increase by 225% in the next decade [1]. Meanwhile, an evolution of the OPSCC treatment paradigm from a primary radiation-based strategy to a primary surgical approach is being considered [2]. Although the epidemiology of this disease has changed and the treatment paradigm is rigorously being evaluated in clinical trials, the diagnostic evaluation of OPSCCs has not been re-examined.

The base of tongue (BOT) is a challenging anatomic subsite of the oropharynx to examine. On transoral visual examination, the BOT is obscured by the oral tongue, “falls vertically away from the view of an examiner,” and even with “a laryngeal mirror or fiberoptic laryngoscope…is still viewed tangentially, making thorough inspection difficult [3].” Examination can further be complicated by a physiologic gag reflex. Clinical examination (intraoral visualization, mirror laryngoscopy and fiberoptic laryngoscopy) can demonstrate the mucosal or exophytic portion of a tumor, suggest an endophytic component (asymmetry) and local tumor extension. Palpation is crucial and may provide a sense of midline extent or be the only indication of a mass that is otherwise not visible. However, the palpable portion of a tumor is likely limited to the exophytic portion of a mass or the superior portion of an endophytic tumor. Therefore, even with the aid of general anesthesia, the detection and the description of BOT masses and their extent can be difficult and is driven by experience [3], [4].

Traditional modalities used to image the oropharynx comprise of positron emission tomography (PET), computed tomography (CT) and magnetic resonance imaging (MRI). PET is a sensitive means of identifying the presence, absence or suggestion of a tumor, but lacks soft tissue and primary tumor delineation, [5] and the physiologic ^18^F-fluorodeoxyglucose (FDG) activity of oropharyngeal lymphoid tissue can be misleading. CT is useful to determine hyoid or mandibular involvement, however, BOT can be obscured by dental artifact [6]. MRI offers improved soft tissue delineation, although small tumors can be difficult to discern from surrounding lingual lymphoid tissues [6]. Furthermore, MRI is a lengthy exam that requires patient cooperation, as well as experienced radiologic interpretation [7]. All three of these imaging methods are cross-sectional, provide static views of the tumor relative to surrounding structures and are expensive. Indeed, radiologists recommend a combination of these three modalities in the initial evaluation of tumor and nodal status to account for the strengths and weaknesses of each modality [7].

Therefore, we were interested in an imaging modality that could be used in the office to visualize BOT lesions. Given the success of ultrasound in depicting oral tongue lesions and predicting their depth [8], [9], we hypothesized that ultrasound could be used to visualize BOT masses in patients with known or suspected BOT malignancy.

## Methods

Study subjects were prospectively enrolled in this investigational review board-approved study (Greater Baltimore Medical Center; #10-048-07). Study participants provided written informed consent. Eligibility criteria included an incident diagnosis of BOT malignancy. Patients who had prior radiation therapy, trismus, and/or previous ablative surgery of the head and neck were ineligible. Cases were either referred by Johns Hopkins Head and Neck Surgery head and neck oncologic surgeons or identified at a weekly multidisciplinary tumor board. Controls were subjects without head and neck cancer. All ultrasound examinations were performed at the Milton J. Dance Jr. Head and Neck Center Greater Baltimore Medical Center (Baltimore, Maryland). Subjects who provided informed consent were included in the study population.

Each subject had a unique study identification number. Clinical records of cases were reviewed to identify tumor subsite, stage, histopathology, clinical examination, treatment plans and imaging results. Based upon this information, a pre-ultrasound clinical impression was recorded. Transcervical ultrasound procedures were performed at the time of study visit by investigators (RGB, CF). A Toshiba ultrasound model SSA-580A was used to examine all subjects. The transducer used was convex (3.75- 6.0 Mega hertz (MHz); Model PVQ-375A) and set at 6 MHz. Uniform anatomic reference points were defined. The landmarks for the BOT were determined by identifying the central portions of the hyoid bone and the mandible and dividing this into thirds. The posterior third was considered the ultrasonographic base of tongue.

Ultrasound examination was performed in a standardized fashion. Subjects were seated in an ENT exam chair. For the midline sagittal view, the probe was placed in the midline between the mental notch and the hyoid bone. Bilateral parasagittal views were obtained approximately 2-cm lateral to submental midline. All sagittal views required that both the hyoid and mandible be visualized. Coronal views were obtained by positioning the transducer above the hyoid bone in an angled fashion (30–45 degrees) from the horizontal plane of the hyoid bone. In the coronal view, the lingual artery and its relationship to the tumor was assessed. In addition, tongue mobility was assessed in the anterior-posterior and medio-lateral axes.

Data were recorded using a standardized case report form at the time of ultrasound examination. An ultrasound impression was ascertained which recorded characteristics of the normal tongue and lesion (if present). The ultrasound findings were compared with relevant clinical parameters.

Clinical data did not provide tumor size in millimeters. To compare clinical size of tumors with ultrasound measurements, we considered AJCC tumor size (i.e. T1 tumors <2 cm, T2 2–4 cm, T3>4 cm etc) [10]. The clinical tumor (T) stage for each study subject was recorded. To generate an ultrasound T stage, we used the largest ultrasound tumor measurement for each study subject and applied AJCC criteria for oropharyngeal tumor size to assign an ultrasound tumor size (T1–T3 or greater). This definition used only size as criteria and therefore T3 and T4 were combined into one category, “T3 or greater” for both.

Study data were collected and managed using REDCap (Research Electronic Data Capture) electronic data capture tools hosted by Johns Hopkins Bloomberg School of Public Health [11]. REDCap is a secure, web-based application designed to support data capture for research studies, providing: 1) an intuitive interface for validated data entry; 2) audit trails for tracking data manipulation and export procedures; 3) automated export procedures for seamless data downloads to common statistical packages; and 4) procedures for importing data from external sources. Data were exported to Stata 12.0 (College Station, Texas) for further analysis.

The primary variable of interest was the presence or absence of a lesion. Descriptive characteristics were summarized. 2×2 tables were created and chi-square tests were used to determine statistical significance except for paired comparisons in which McNemar’s test was implemented.

## Results

Twenty-two cases with known or suspected base of tongue cancers and 18 controls were enrolled in this study. The majority of the cases were white (19 of 22, 86.4%) and male (95.5%) with early tumor stage and nodal disease that was stage 2a or greater. Median age was 60 years (range 54, 68.3). All 22 cases were histopathologically confirmed as malignant (20 SCC, 1 adenocarcinoma and 1 mucoepidermoid carcinoma).

All study subjects completed the ultrasound examination which was performed by a head and neck surgeon (RB and CF). Still images and data were reviewed by both head and neck surgeons prospectively and a radiologist retrospectively (SS).

### Ultrasound Examinations

**Figure 1:**
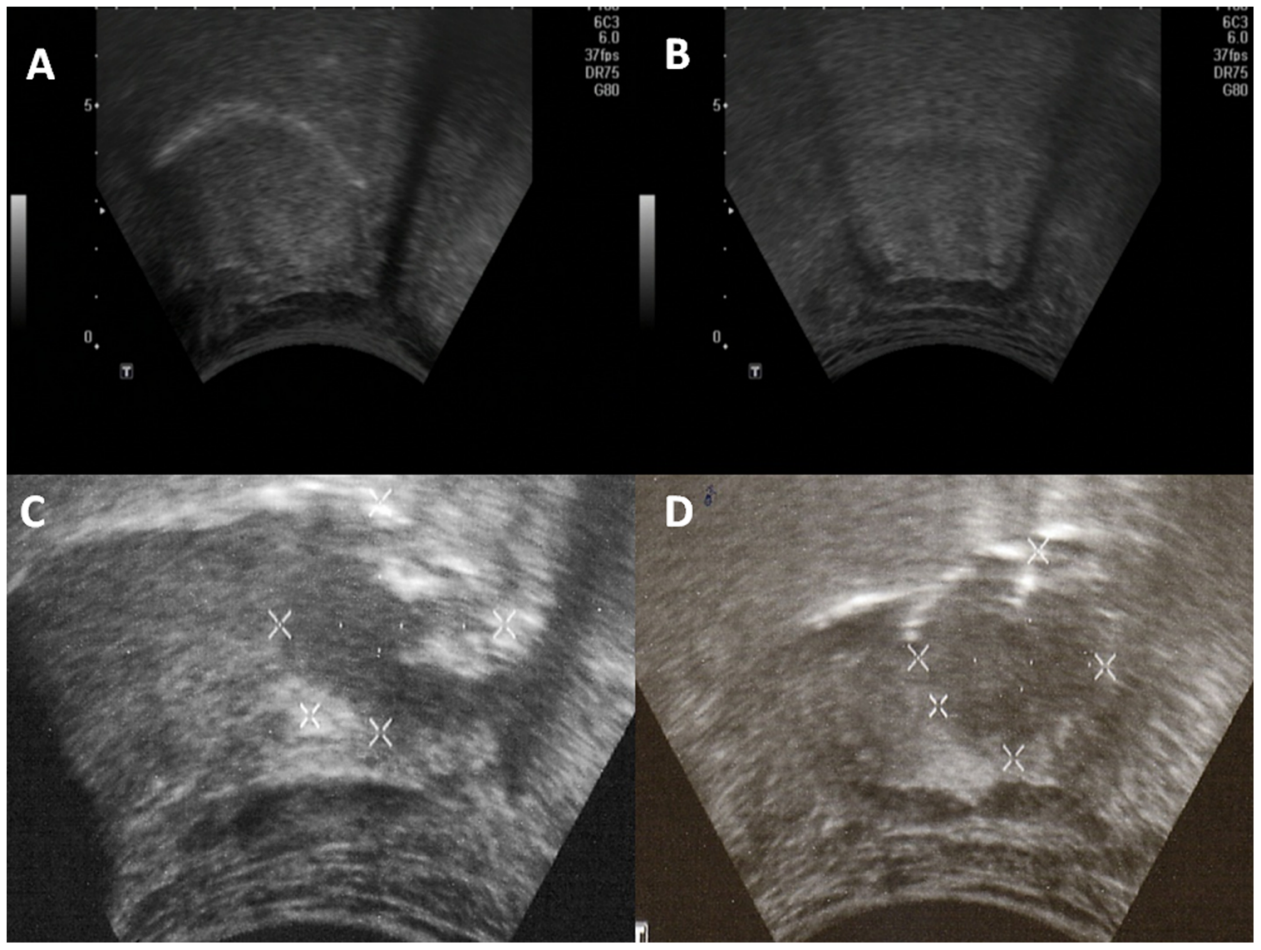
The transcervical ultrasound appearance of the normal BOT is shown in parasagittal (A) and coronal views (B). A BOT tumor is shown in parasagittal (C) and coronal views (D). A large endophytic portion as well as a superficial mucosal component are observed in both views. On coronal view, the relationship with midline is appreciated. The distance between each calibration bar is 10 mm.

22 of 22 clinically suspicious BOT tumors were visualized by ultrasound. The normal base of tongue in the majority of cases with suspected malignancy was isoechoic with heterogeneous foci (21 of 22, 95.5%). Similarly, the base of tongue of all 18 controls was described as isoechoic with heterogeneous foci (100%, Figure 1a–b). There were no suspicious lesions identified on ultrasound examination in any of the controls.

The ultrasound characteristics of BOT tumors are summarized (Table 1) and depicted in Figure 1. The majority of suspicious lesions were hypoechoic (20 of 22, 90.9%). Relative to the lesions, the margins were either hypoechoic (11 of 22, 50%) or isoechoic (10 of 22, 47.6%) and were mostly irregularly shaped (21 of 22, 95.5%). The margins of lesions were rarely observed to be well-circumscribed (3 of 22, 13.6%), but rather intermediate (19 of 22, 81.8%).

**Table 1:** Ultrasonographic characteristics of base of tongue lesions.

	n (%)
**Echogenicity of normal tongue**	
Isoechoic with heterogeneous foci	21 (95.5)
Homogeneous	1 (4.6)
**Base of tongue lesion visualized**	
Yes	22 (100)
No	0 (0)
**Echogenicity of lesion**	
Hypoechoic	20 (90.9)
Isoechoic with heterogeneous foci	2 (9.1)
**Echogenicity of margin**	
Isoechoic with/without heterogeneous foci	10 (47.6)
Hypoechoic	11 (50.0)
Other	1 (4.6)
**Shape of margin**	
Regular	1 (4.6)
Irregular	21 (95.5)
**Clarity of margin**	
Well-circumscribed	3 (13.6)
Intermediate	19 (81.8)
Unclear	1 (4.6)
**Adjacent anatomic site involvement**	
Yes	5 (22.7)
No	17 (77.3)
**Lesion crosses midline**	
Yes	10 (45.5)
No	12 (54.6)
**Lingual artery visualized**	
Yes	22 (100)
No	0 (0)
**Mean distance from lesion to lingual artery in mm (range)**	6.6 (0–17.8)
**Endophytic vs. exophytic extent of lesion**	
Endophytic	0
Exophytic	7 (31.8)
Mixed	15 (68.2)
**Median tumor size in mm (range)**	
Superior-inferior	26.4 (10.4–43.4)
Medial-lateral	25.3 (5.4–48.5)
Anterior-posterior	27.4 (9.7–46.3)

**Figure 2:**
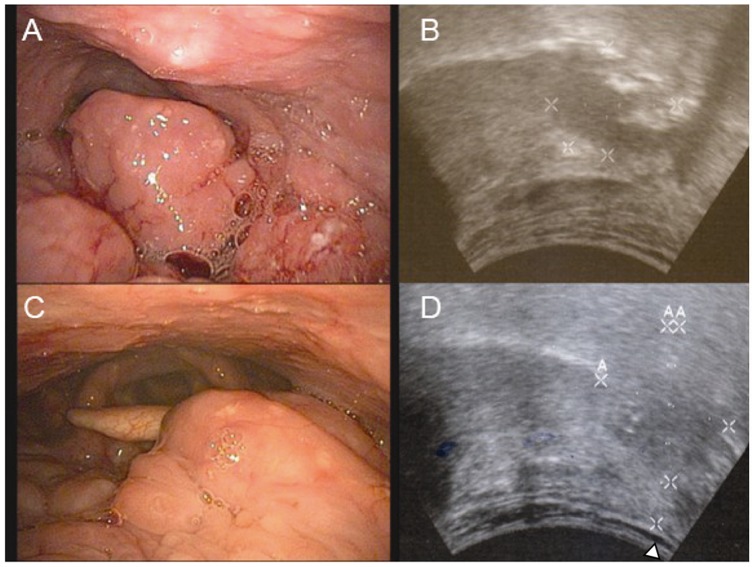
Comparison of fiberoptic BOT examination and ultrasound. Panel A shows the fiberoptic image of a BOT lesion which appears exophytic and ulcerative. On ultrasound parasagittal view (B), a large endophytic portion which is hypoechoic relative to the remainder of the tongue is revealed (marked by x’s) in addition to the known exophytic portion. Panel C shows fiberoptic image of a BOT with asymmetry and obvious bulge which is consistent with a clinically exophytic tumor. On ultrasound (panel D) an endophytic component is appreciable and is 7 mm from the geniohyoid muscle (). The distance between each calibration bar is 10 mm.

Tumor extent and relationship with surrounding anatomy was described by ultrasound. In almost half of the cases, the lesions were observed to cross midline (10 of 22, 45.5%). On coronal view, the ipsilateral lingual artery was visualized in all 22 cases. The mean distance of the lingual artery from the lateral margin of the lesion was 6.6 mm (range 0, 17.8). When considering whether the masses were exophytic or endophytic, the majority on ultrasound appeared to be a combination of exophytic and endophytic (15 of 22, 68.2%). The remainder appeared to be purely endophytic (7 of 22 31.8%) (Figure 2). In five cases (22.7%), the tumors were noted to have adjacent site involvement. Three tumors extended to the ipsilateral tonsil, one involved ipsilateral vallecula and another involved both the ipsilateral tonsil as well as the vallecula.

Three-dimensional measurements of each lesion were summarized (table 1). The superior-inferior and anterior-posterior dimensions were measured on parasagittal view, while the width (medio-lateral) was appreciated on coronal view. The smallest tumor measurements in each dimension ranged between 5.4 and 10.4 mm. Median tumor measurements in all three dimensions were between 25 and 27 millimeters.

Tongue mobility was assessed in all subjects. 100% of subjects were determined to have full anterior and lateral excursion.

### Comparison of Ultrasound and Clinical Examinations

BOT clinical and ultrasound examinations were compared (Table 2). To compare clinical and ultrasound size, we compared tumor stage. Most tumors were clinically staged T2 or greater (16 of 22, 72.7%). Using a similar staging system for ultrasound, most tumors were ≥T2 (19 of 22, 86.4%). Although there was no statistical difference between the clinical and ultrasound staging (p = 0.41), ultrasound staging was more right-skewed, when compared with clinical staging (i.e. ultrasound staging provided larger tumor stage than clinical staging).

**Table 2:** Clinical and ultrasound characteristics of base of tongue lesions.

	Clinical	Ultrasound
**Tumor staging**		
T1	6	3
T2	11	14
T3 and T4	5	5
**Crosses midline**		
Yes	7	10
No	15	12
**Endo- vs. exophytic**		
Exophytic	13	0
Endophytic	9	7
Mixed	0	15
**Adjacent site involvement**		
Yes	5	5
No	17	17
**Description of lesion on PET**		
Mass	16	22
Asymmetry	4	0
No lesion identified	2	0

Tumors were observed to cross midline in 7 of 22 (31.8%) clinical exams and 10 of 22 (45.5%) ultrasound exams. On clinical examination, 5 of 22 (22.7%) tumors were noted to have adjacent site involvement. Similarly, 5 of 22 tumors on ultrasound were deemed to have either tonsillar or vallecular involvement. However, the five tumors with adjacent site involvement on clinical and ultrasound examination were not the same. Three of five had concordant assessments made on clinical and ultrasound examinations.

On clinical examination tumors were classified as either exophytic (13 of 22, 59.1%) or endophytic (9 of 22, 40.9%). Meanwhile, on ultrasound, the majority of tumors were a mixture of endophytic and exophytic (15 of 22, 68.2%) or purely endophytic (7 of 22, 31.8%). (Figure 2).

A primary lesion was evident on PET scan in 16 of 22 cases. However, in 6 of 22 (27%) cases, the lesions were reported to be “asymmetries” or no lesion was identified. By contrast all 22 lesions were visualized on ultrasound.

## Discussion

To our knowledge this is the first study to comprehensively and prospectively evaluate the ultrasound characteristics of base of tongue tumors. All 22 (100%) clinically suspicious lesions were visualized by transcervical ultrasound. Normal BOT in both cases and controls was isoechoic with heterogeneous foci. BOT lesions were uniformly hypoechoic with irregular and intermediate margins and therefore could readily be distinguished from the surrounding normal tongue base musculature.

Initial reports in 1970s and 1980s suggested that ultrasound could be used to identify BOT lesions [12]. However, these limited reports did not describe the appearance of tumors, included tumors of heterogeneous anatomic sites (oral tongue, base of tongue, floor of mouth), were small in sample size and retrospective [9], [12], [13]. Normal BOT anatomy has previously been described in the context of obstructive sleep apnea and speech [14], [15]. Additionally, the feasibility of fine needle aspirations of the normal BOT (and floor of mouth) has recently been performed in cadavers [16]. In this study, patients with BOT malignancies underwent ultrasound examination using a uniform protocol in a prospective fashion.

In the clinical assessment of BOT tumors, size, extent across midline, adjacent site involvement, laterality, relationship with neurovascular structures and the three-dimensional nature are important features for staging and for determination of eligibility for surgical vs. non-surgical primary therapeutic modalities. Yet, clinical examination of the tongue base is challenging and may not be informative regarding these clinically significant characteristics, even in the setting of anesthesia and with the aid of illuminated rigid endoscopy [3]. Ultrasound may help overcome these limitations. It presents a tool to visualize BOT tumors transcervically in awake patients in an office-based setting and is virtually risk-free (non-ionizing radiation) and repeatable. In this study, ultrasound could be used to assess tumor size, extent of tumor with respect to midline, endophytic and exophytic tumor extent, relationship with the lingual artery, adjacent site involvement and view the tumor in three-dimensions in real time, all of which are features not always ascertainable on clinical or radiographic examination.

From ultrasound we appreciate that the majority of base of tongue tumors indeed have an endophytic component. This finding makes sense since oropharynx tumors are thought to arise from the reticulated epithelium of cryptic lymphoid tissue [17]. Therefore, although an endophytic component is not always appreciable by clinical examination, ultrasound permits the examiner to see the endophytic portion and its inferior extent both in sagittal view and in real time. In addition, the extent of the endophytic portion across midline is readily visualized on coronal view. Although of clinical significance, the midline extent of a tumor, specifically its endophytic portion can be difficult to ascertain clinically. The midline on clinical examination (in-office or operative) is imprecise and somewhat arbitrary, and the endophytic portion of a tumor is not necessarily appreciable clinically. Ultrasound permits an anthropomorphic midline to be designated while providing visualization of the endophytic portion. Indeed, our data indicate that ultrasound detected a ∼14% increase in the number of tumors crossing midline as compared to clinical examination. The specificity of this data remains to be determined.

As compared with other radiographic modalities, ultrasound offers significant advantages. BOT tumors can be difficult to identify on CT because of artifact and poor soft tissue definition, however on ultrasound BOT lesions are distinct from the surrounding normal tongue base, even for T1 lesions. When lesions are visualized by CT, the endophytic component and midline extent, both of which can be determined by ultrasound, at times cannot be ascertained by CT as the plane between tumor and normal BOT appears contiguous. Soft tissue delineation on MRI is better than CT, but small tumors can still be difficult to discern from the surrounding lymphoid tissue [7]. By contrast, ultrasound can demonstrate an alteration in the normal symmetric distribution of lymphoid tissue. PET scans have inherent limitations in the diagnostic evaluation of BOT cancers due to the physiologic uptake of glucose in the lingual lymphoid tissue and are therefore not considered helpful in primary tumor staging [7]. Ultrasound provided visualization of tumors that were not reported as distinct lesions on PET scan in 27% of cases (6 of 22). These characteristics of BOT tumors and their extent that can be appreciated by ultrasound and not by other radiographic modalities can have significant implications for radiotherapy planning and can be informative for surgical planning.

With the introduction of transoral surgery the evaluation of BOT tumors and their resectability has increased in importance. Similar to ultrasound evaluation of thyroid, ultrasound of the BOT overcomes the limitations of clinical examination and provides improved understanding of the tumor and its characteristics spatially in the oropharynx. Therefore, ultrasound may be of significance in operative planning. For example, ultrasound may help to determine the resectability based upon the endophytic extent that would otherwise not be appreciable. Based on the size and location of a BOT tumor and adding the necessary margins to achieve oncologic control, a surgeon can preoperatively estimate the defect and determine the feasibility of achieving negative margins. Ultrasound may also be used to direct a lingual tonsillectomy to the location of a small primary tumor that otherwise would have required a larger extent of excision and potential morbidity. Furthermore, its relationship relative to the lingual artery is of surgical relevance. These results are compelling and provide the basis for a future study to evaluate the role of ultrasound in preoperative planning to determine its ability to achieve negative margins and accurately predict tumor extent. It must be noted that our protocol was performed in upright patients and acknowledge that supine positioning is more applicable to operative planning.

This study has significant potential screening implications. As the incidence of BOT cancers rises [1], [18], the impetus for identifying a screening tool increases. We have previously demonstrated that a Pap-smear equivalent for the oropharynx is not feasible, largely due to the poor visualization of the oropharynx, and the anatomic considerations of the BOT which render sampling difficult [19]. Therefore, ultrasound may circumvent prior visualization issues and provide a promising modality to direct a screening tool to a specific lesion of concern.

It is worth noting that there was a potential for bias in our study. We performed ultrasound examinations with the knowledge that subjects did or did not have a clinically suspicious malignancy. However, a blinded study would not have been possible as this study was largely exploratory, designed to visualize BOT lesions and their characteristics. Prior to this study, the literature lacked an ultrasonographic description of BOT malignancies. While this study was not designed to evaluate the diagnostic ability of ultrasonography for BOT tumors, it is important to note that in radiographic clinical practice, knowledge of clinically relevant information is encouraged to improve the odds of a proper diagnosis [20]. Lastly, it is important to recognize that despite the promising anatomic information provided by ultrasound, at this time it does not replace clinical and operative examination of the BOT.

Ultrasonography is a promising modality that may play a role in future diagnostic, staging, operative and potential screening evaluations of BOT malignancies. Future studies are required to compare transcervical ultrasonography of the BOT with conventional imaging modalities (including CT and MRI with contrast).
